# CEMIP upregulates BiP to promote breast cancer cell survival in hypoxia

**DOI:** 10.18632/oncotarget.27036

**Published:** 2019-07-02

**Authors:** Anna Banach, Ya-Ping Jiang, Eric Roth, Cem Kuscu, Jian Cao, Richard Z. Lin

**Affiliations:** ^1^ Molecular and Cellular Biology Program, Stony Brook University, Stony Brook, NY, USA; ^2^ Department of Physiology and Biophysics, Institute of Molecular Cardiology, Stony Brook University, Stony Brook, NY, USA; ^3^ Medical Scientist Training Program, Stony Brook University, Stony Brook, NY, USA; ^4^ Transplant Research Institute, University of Tennessee Health Science Center, Memphis, TN, USA; ^5^ Division of Cancer Prevention, Department of Medicine, Stony Brook University, Stony Brook, NY, USA; ^6^ Medical Service, Northport Veterans Affairs Medical Center, Northport, NY, USA

**Keywords:** CEMIP, BiP, hypoxia, autophagy

## Abstract

Cell migration-inducing protein (CEMIP) and binding immunoglobulin protein (BiP) are upregulated in human cancers, where they drive cancer progression and metastasis. It has been shown that CEMIP resides in the endoplasmic reticulum (ER) where it interacts with BiP to induce cell migration, but the relationship between the two proteins was previously unknown. Here we show that CEMIP mediates activation of the BiP promoter and upregulates BiP transcript and protein levels in breast cancer cell lines. Moreover, CEMIP overexpression confers protective adaptations to cancer cells under hypoxic conditions, by decreasing apoptosis, activating autophagy, and increasing glucose uptake, to facilitate tumor growth. We demonstrate that BiP signals downstream of CEMIP, modulating cellular resistance to hypoxia. Reducing BiP in CEMIP-expressing cells sensitized cells to hypoxia treatment, decreased glucose uptake, and resulted in tumor regression *in vivo*. Our study provides insights into the link between CEMIP and BiP expression and the pro-survival role they play in hypoxia. Better understanding of the mechanisms behind cancer cell adaptations to harsh tumor environments could lead to development of improved cancer treatments.

## INTRODUCTION

CEMIP is a poorly characterized protein that is highly expressed in human cancers and associated with poor patient survival [1–5]. Several reports have characterized CEMIP as a key protein involved in cancer progression due to its ability to promote proliferation, migration, hyaluronic acid depolymerization, and epithelial-to-mesenchymal transition (EMT) [1–3, 6–15]. A previous study has identified CEMIP as residing in the ER, where it binds to a major chaperone, BiP, resulting in increased cytosolic calcium levels. This further leads to protein kinase C alpha activation, which triggers its downstream targets, including ERK1/2, resulting in enhanced cell migration [1]. More recent reports have shown that CEMIP might mediate an anti-apoptotic role; however, the mechanism by which it exerts this function is still not clearly understood [8, 16].

Hypoxia and nutrient deprivation are common challenges that cancer cells must overcome in order to survive in the harsh tumor microenvironment. Understanding the molecular mechanisms that underlie these adaptations could lead to development of more effective cancer therapies. It was previously shown that CEMIP is induced by nuclear factor kappa-light-chain-enhancer of activated B cells (NF-κB), as well as by hypoxic conditions *via* hypoxia-inducible factor-2α (HIF-2α), suggesting it might serve a protective function in response to stress [6, 17]. Other reports have shown that CEMIP can suppress apoptosis *via* epidermal growth factor receptor (EGFR) signaling as well as by enhancing glycogen breakdown to promote cancer cell survival [8, 16]. Therefore, it is not unlikely that CEMIP serves a protective function under the hypoxic conditions within the tumor environment.

CEMIP forms a stable complex with BiP in the ER, leading to enhanced cell migration [1]. BiP is an ER resident chaperone that binds to proteins to stabilize them and assist in proper folding [18]. In addition to its canonical function in the ER, BiP was also found to play a critical role in cancer progression by promoting cancer cell survival, proliferation, migration, and chemoresistance [19–25]. Other reports indicate that BiP is induced in cancer cells in response to hypoxia and serves a protective function by means of activating autophagy [18, 19, 22]. Autophagy is one of the survival mechanisms in response to stress, including oxygen deficit, by which cells recycle cytoplasm and organelles in order to generate energy and nutrients. During this process, numerous autophagy-related proteins, including LC3, participate in the formation of autophagosomes. These double layer membrane vesicles enclose cellular components and then fuse with lysosomes, whose digestive enzymes degrade the cargo [26].

Based on these collective findings, we hypothesized that CEMIP promotes cell survival in hypoxic conditions by upregulating BiP expression. In this study, we show that CEMIP upregulates BiP at the transcriptional level, which leads to decreased apoptosis and increased autophagy under oxygen deficit. Identifying the correlation between CEMIP and BiP expression as well as the protective functions that they provide to cancer cells exposed to hypoxia could lead to the development of more efficient chemotherapeutics.

## RESULTS

### CEMIP and BiP expression are correlated in human breast cancer cell lines

**Figure 1:**
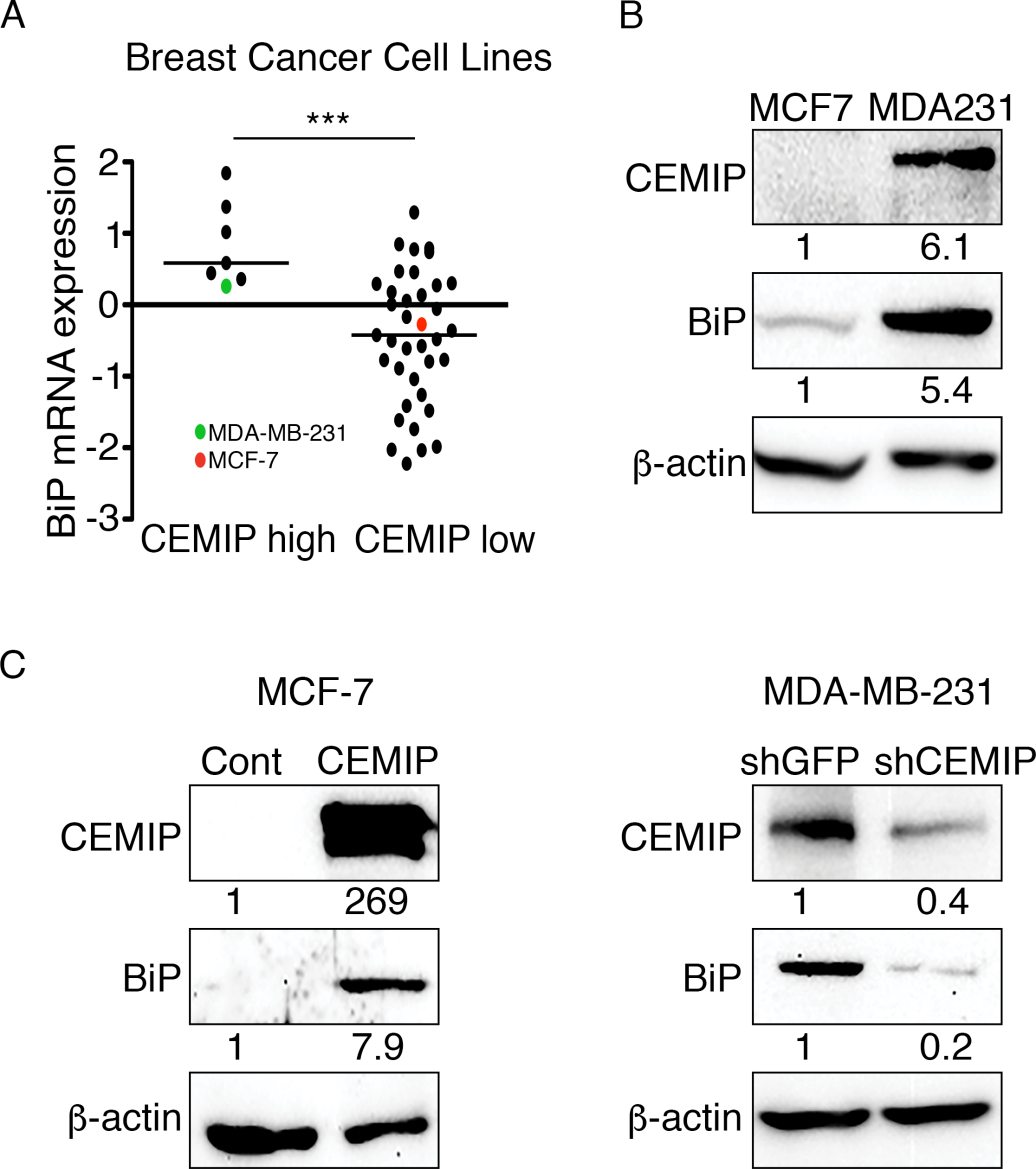
CEMIP upregulates BiP in human breast cancer cell lines. **(A)** mRNA expression z-scores for 51 breast cancer cell lines contained in the Cancer Cell Line Encyclopedia (Novartis/Broad, Nature 2012) were analyzed using cBioPortal. BiP mRNA expression in cell lines with high (z-score > 0.6) or low (z-score < -0.3) CEMIP mRNA levels is plotted. Indicated in red are MCF-7 and in green are MDA-MB-231 cells. Bars indicate median. ^***^p < 0.001, Student’s t-test. **(B)** A western blot of MCF-7 (MCF7) and MDA-MB-231 (MDA231) total cell lysates was probed with the indicated antibodies. β-actin was used as a loading control. Densitometric analysis was performed to quantify protein bands. CEMIP and BiP levels were normalized to β-actin. **(C)** Lysates of (left) MCF-7 cells stably expressing CEMIP or empty vector as a control (Cont) and (right) MDA-MB-231 cells stably expressing shRNA against CEMIP (shCEMIP) or GFP as a control (shGFP) were subjected to western blotting using the indicated antibodies. β-actin was used as a loading control. Densitometric analysis was performed to quantify protein bands. CEMIP and BiP levels were normalized to β-actin.

CEMIP and BiP are overexpressed in cancers, where they contribute to cancer progression and metastasis [1–5, 20, 22–24]. It has been shown that CEMIP forms a stable complex with BiP in the ER, leading to enhanced cell migration [1]; however, the relationship between the two proteins remains poorly understood. To investigate a possible link between CEMIP and BiP expression, we analyzed mRNA expression in 51 breast cancer cell lines characterized in the Cancer Cell Line Encyclopedia (Novartis/Broad, Nature 2012) using cBioPortal [27, 28]. Surprisingly, the median mRNA level of BiP was higher in cell lines with high CEMIP mRNA levels (z-score > 0.6) than in cell lines with low CEMIP expression (z-score < -0.3) (Figure 1A). This result led us to hypothesize that there is a relationship between the expression of CEMIP and BiP. We chose two cell lines from Figure 1A—low CEMIP-expressing MCF-7 and high CEMIP-expressing MDA-MB-231—to investigate this possibility. Western blotting revealed that MCF-7 cells express low levels of the CEMIP and BiP proteins relative to MDA-MB-231 cells, in agreement with the mRNA data (Figure 1B). Stable overexpression of CEMIP in MCF-7 cells (to produce cells referred to as MCF-7 CEMIP) was found to increase the level of BiP protein as compared to the control cell line stably expressing empty vector (referred to as MCF-7 Cont cells) (Figure 1C). Conversely, MDA-MB-231 cells stably expressing an shRNA to silence CEMIP expression (referred to as MDA-MB-231 shCEMIP cells) exhibited decreased BiP protein levels as compared to control MDA-MB-231 cells stably expressing shGFP (referred to as MDA-MB-231 shGFP cells) (Figure 1C). Next, we determined the effects of transient overexpression of CEMIP on BiP protein levels. Transient expression of CEMIP in MCF-7 cells resulted in increased BiP levels (Supplementary Figure 1) as compared to empty vector control, further substantiating our findings. In addition, the levels of two ER chaperone proteins, Calnexin and PDI, remained unchanged between CEMIP and empty vector-expressing MCF-7 cells (Supplementary Figure 1), indicating that CEMIP-mediated BiP upregulation is not due to a generalized cellular response. To further ensure that CEMIP overexpression leading to increased BiP levels is not an artifact, we transiently expressed CEMIP alongside with other exogenous proteins, small protein tissue inhibitor of metalloproteinase-1 and large protein EGFR, in MCF-7 cells. The results showed that BiP upregulation was caused by the overexpression of CEMIP but not the other proteins (Supplementary Figure 1). Lastly, we chose an additional low CEMIP-expressing breast cancer cell line from Figure 1A, BT-474, to infect with adenoviruses expressing CEMIP-GFP or GFP as a control. Adenoviral overexpression of CEMIP in BT-474 cells led to increased BiP protein levels (Supplementary Figure 2). Taken together, these results suggest that CEMIP upregulates BiP expression in breast cancer cell lines.

### CEMIP mediates activation of the BiP promoter

**Figure 2:**
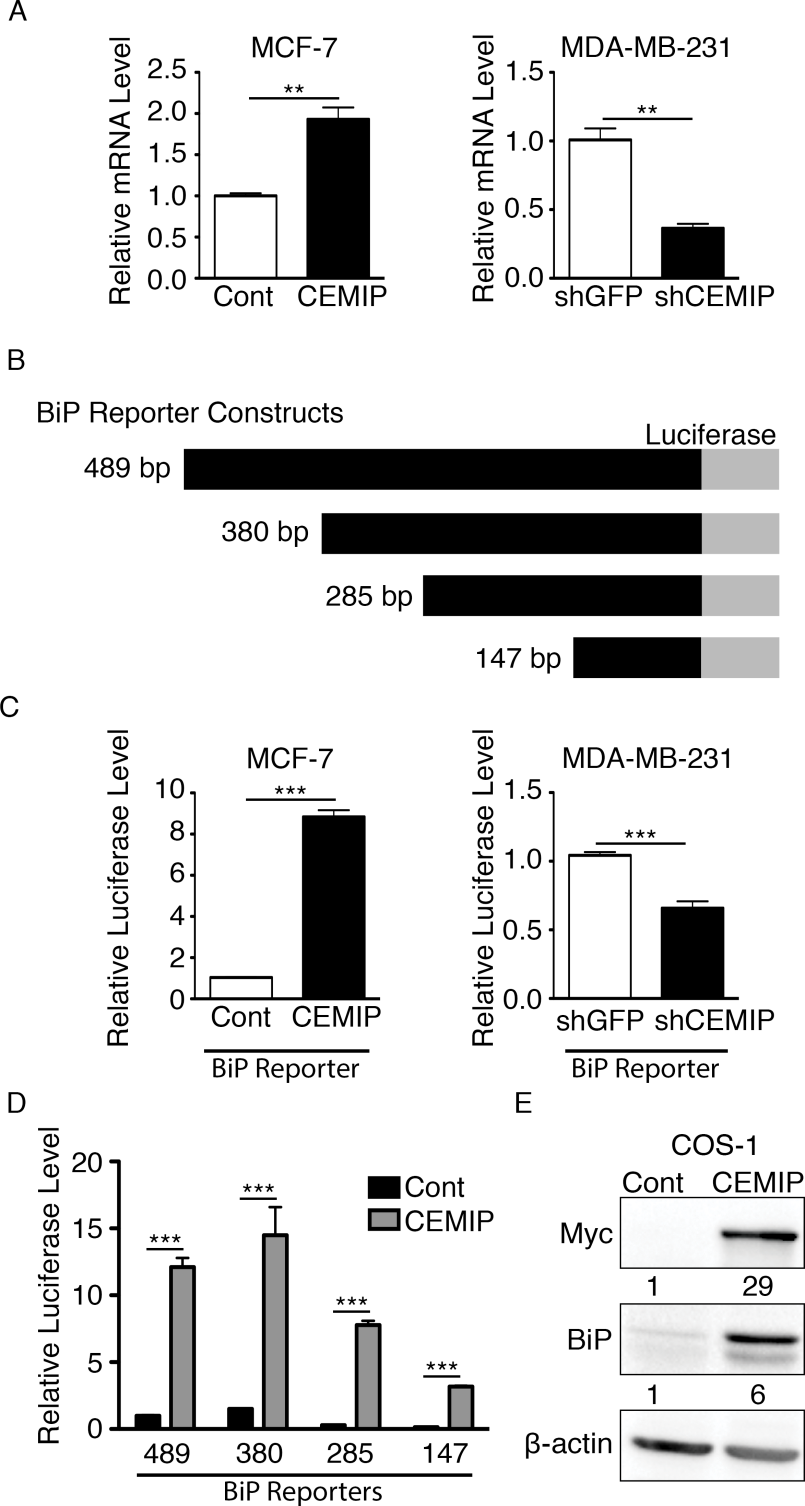
CEMIP upregulates BiP transcription through a minimal BiP promoter region. **(A)** Relative BiP mRNA levels as determined by qRT-PCR in (left) MCF-7 Cont or MCF-7 CEMIP cells and (right) MDA-MB-231 shGFP or MDA-MB-231 shCEMIP cells. Mean ± SEM, **p < 0.01, Student’s t-test. BiP expression was normalized to HPRT-1. **(B)** A schematic representation of BiP reporter constructs. The 489 bp BiP promoter sequence cloned into the firefly luciferase reporter plasmid and reporter plasmids with promoter sequences of the indicated lengths produced by 5’ truncations. **(C)** Relative firefly luciferase reporter activity in (left) MCF-7 Cont or MCF-7 CEMIP cells and (right) MDA-MB-231 shGFP or MDA-MB-231 shCEMIP cells transfected with the 489 bp human BiP promoter reporter construct. Firefly luciferase reporter activity was normalized to the *Renilla* luciferase signal. Mean ± SEM, ^***^p < 0.001, Student’s t-test. **(D)** Cos-1 cell were co-transfected with different BiP reporter constructs indicated in B) and either CEMIP or empty vector as a control (Cont). Firefly luciferase reporter activity driven by each BiP promoter construct was normalized to the *Renilla* luciferase signal. Mean ± SEM, ^***^p < 0.001, Student’s t-test. **(E)** Cos-1 cells were transfected with CEMIP or empty vector (Cont). A western blot of whole cell lysates was probed with the indicated antibodies. Anti-Myc antibodies recognize the Myc tag on CEMIP. β-actin was used as a loading control. Densitometric analysis was performed to quantify protein bands. CEMIP and BiP levels were normalized to β-actin.

To determine if CEMIP regulates BiP transcription, we performed qRT-PCR to quantify relative BiP mRNA levels in our modified MCF-7 and MDA-MB-231 cell lines. MCF-7 CEMIP cells showed significantly higher BiP mRNA levels than MCF-7 Cont cells (Figure 2A). Conversely, MDA-MB-231 shCEMIP cells showed significantly reduced BiP expression as compared to control MDA-MB-231 shGFP cells (Figure 2A). We next examined the effect of CEMIP on BiP promoter activity. Using a 489 bp human BiP promoter sequence linked to a firefly luciferase reporter gene (see Figure 2B), we found that BiP promoter activity is significantly higher in MCF-7 CEMIP cells than in MCF-7 Cont cells, whereas MDA-MB-231 shCEMIP cells have significantly reduced BiP promoter activity as compared to MDA-MB-231 shGFP cells (Figure 2C). To determine the region within the BiP promoter that confers CEMIP-mediated activation, four BiP reporter constructs of different lengths depicted in Figure 2B were co-transfected with CEMIP or control vector into COS-1 cells. Luciferase assays showed that overexpression of CEMIP significantly increased the activity of all four reporter constructs, indicating that CEMIP upregulates BiP transcription within a minimal promoter region (Figure 2D). Lastly, we confirmed that CEMIP overexpression increased BiP protein levels in COS-1 cells (Figure 2E).

### CEMIP reduces apoptosis and promotes autophagy under hypoxia

**Figure 3:**
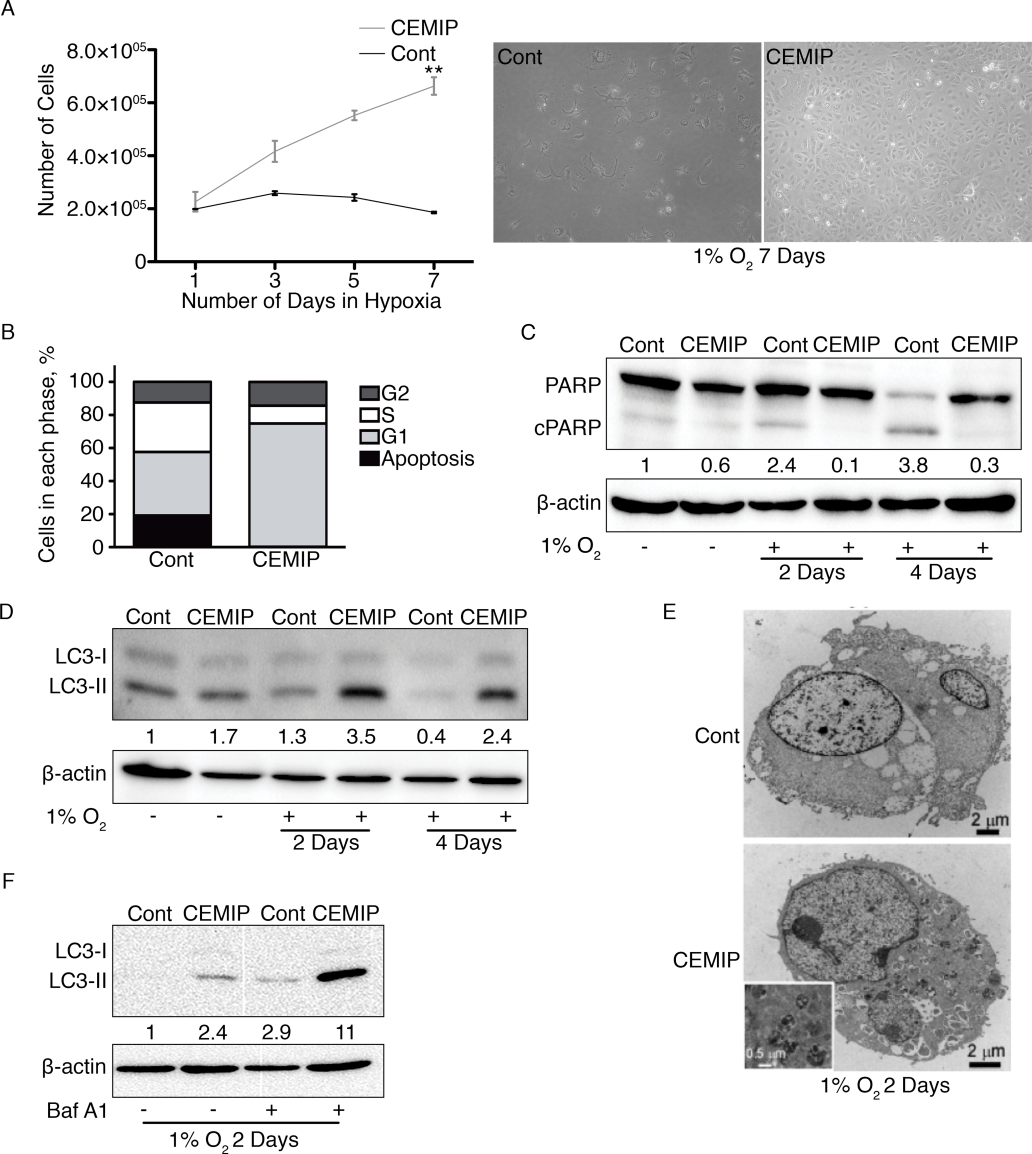
CEMIP promotes cell survival in hypoxic conditions by decreasing apoptosis and increasing autophagy. MCF-7 Cont and MCF-7 CEMIP cells were cultured under normoxic or hypoxic (1% O_2_) conditions as indicated. **(A)** 180,000 cells were plated, placed in hypoxic conditions, and counted on days 1, 3, 5, and 7. 10X bright field images of cells exposed to 1% O_2_ for 7 days are shown on the right. Mean ± SEM, **p < 0.01, Student’s t-test performed for Day 7. **(B)** Cells exposed to 1% O_2_ for 6 days were stained with propidium iodide and subjected to flow cytometry. The percentage of apoptotic cells and cells in the G2, S and G1 phases of the cell cycle were determined from the flow cytometry histograms. **(C)** Lysates of cells grown in normoxia or exposed to hypoxia for 2 or 4 days were subjected to western blotting. The anti-PARP antibody detects full-length and cleaved PARP (cPARP). β-actin was used as a loading control. Densitometric analysis was performed to quantify protein bands. Cleaved PARP levels were normalized to β-actin. **(D)** Western blot analysis of cells treated as in C. The anti-LC3 antibody recognizes LC3-I and LC3-II. β-actin was used as a loading control. Densitometric analysis was performed to quantify protein bands. LC3-II levels were normalized to β-actin. **(E)** Transmission electron microscopy images of cells exposed to hypoxia for 2 days. The inset shows a higher magnification image of autophagosomes in MCF-7 CEMIP cells. **(F)** Autophagic flux assay. Cells exposed to hypoxia for 2 days were treated with or without bafilomycin A1 for the last 6 hours. A western blot of cell lysates was probed with anti-LC3 and anti-β-actin (loading control) antibodies. Densitometric analysis was performed to quantify protein bands. LC3-II levels were normalized to β-actin.

A previous study showed that hypoxia induces CEMIP expression in cancer cells *via* direct binding of HIF-2α to a hypoxia response element in the CEMIP promoter [6]. Recent reports showed that CEMIP can suppress apoptosis *via* EGFR signaling as well as by enhancing glycogen breakdown to promote cancer cell survival [8, 16]. Therefore, it is possible that CEMIP expression confers a protective effect under hypoxic conditions. To test this idea, we cultured MCF-7 CEMIP and MCF-7 Cont cells in 1% O_2_ and counted cell numbers over seven days. The results show that MCF-7 CEMIP cells are highly resistant to this hypoxic condition: they continued to grow, whereas the control cell line ceased proliferating (Figure 3A). Next, we exposed the cells to 1% O_2_ for 6 days and then performed cell cycle analysis by flow cytometry (Supplementary Figure 3). Most of the MCF-7 Cont cells were in the S (29.99%) and G1 (38.38%) phases of the cell cycle, and a substantial proportion (19.24%) exhibited a hypodiploid DNA content indicative of apoptosis (Figure 3B). By contrast, the majority of MCF-7 CEMIP cells were in G1 (74.61%), and few apoptotic cells were detected (Figure 3B). We also determined the level of apoptosis by visualizing cleaved PARP on western blots. MCF-7 Cont cells showed a loss of full length PARP and accumulation of cleaved PARP after 4 days of hypoxia treatment, whereas PARP cleavage was minimal in MCF-7 CEMIP cells (Figure 3C). Additionally, we examined PARP cleavage by western blotting in our genetically engineered MDA-MB-231 cells after exposure to hypoxia for 4 days. MDA-MB-231 shCEMIP cells showed increased levels of cleaved PARP as compared to MDA-MB-231 shGFP cells, indicating that CEMIP knockdown sensitized these cells to hypoxia (Supplementary Figure 4).

Autophagy is a survival mechanism by which cells recycle cytoplasm and organelles in order to generate energy in response to stress, including oxygen deficit [19, 26]. During the process of autophagosome formation, LC3-I is lipidated to produce LC3-II [19, 26]. Therefore, we examined LC3-II on western blots to determine if CEMIP expression affects autophagy during exposure to hypoxia. MCF-7 CEMIP cells showed increased expression of LC3-II after 2 days and 4 days of hypoxia treatment as compared to MCF-7 Cont cells (Figure 3D), suggesting that autophagy is increased in CEMIP-expressing cells. To further support this conclusion, autophagosomes in cells exposed to hypoxia for 2 days were visualized by transmission electron microscopy (TEM). We found that only MCF-7 CEMIP cells showed an accumulation of autophagosomes (Figure 3E), further substantiating the hypothesis that CEMIP promotes autophagy during oxygen deficit. A common method used to show autophagic activity is the autophagic flux assay, in which LC3-II protein accumulates when bafilomycin A1 is used to block the fusion of autophagosomes with lysosomes. We performed autophagic flux assays on MCF-7 CEMIP and MCF-7 Cont cells exposed to 1% O_2_ for 2 days. The accumulation of LC3-II in the presence of bafilomycin A1 was much larger in MCF-7 CEMIP cells than in the control cell line (Figure 3F). Together, these data indicate that CEMIP promotes an increase in autophagy in cells exposed to hypoxia. To confirm that CEMIP still upregulates BiP transcription in hypoxic conditions, we determined BiP mRNA levels and BiP promoter activity in modified MCF-7 cells after 2 days of hypoxia treatment. As expected, MCF-7 CEMIP cells showed increased BiP mRNA levels and promoter activity as compared to MCF-7 Cont cells (Supplementary Figure 5).

### CEMIP mediates some of its pro-survival effects by upregulating BiP expression

BiP has been shown to play a critical role in cancer progression by promoting cancer cell survival and chemoresistance [21, 22, 25]. It was also reported that BiP levels increase in response to hypoxic conditions, and upregulation of BiP concomitant with activation of the PERK and IRE1 arms of the unfolded protein response (UPR) is critical for induction of Atg genes, leading to autophagy [18, 19, 22]. First we wanted to establish the difference in activation of the IRE1 and PERK branches of the UPR between MCF-7 CEMIP and MCF-7 Cont cells exposed to hypoxia. After 2 days of hypoxia treatment, we analyzed the levels of phospho-IRE, BECN1, and phospho-eIF2α in these cells by western blotting. We found that CEMIP overexpression led to higher phospho-IRE and its downstream signaling target, BECN1, protein levels as compared to control, indicating increased activity of the IRE1 branch (Supplementary Figure 6). MCF-7 CEMIP cells also showed increased activation of the PERK branch as compared to MCF-7 Cont cells, indicated by higher protein levels of phospho-eIF2α, a downstream target of phospho-PERK (Supplementary Figure 6).

**Figure 4:**
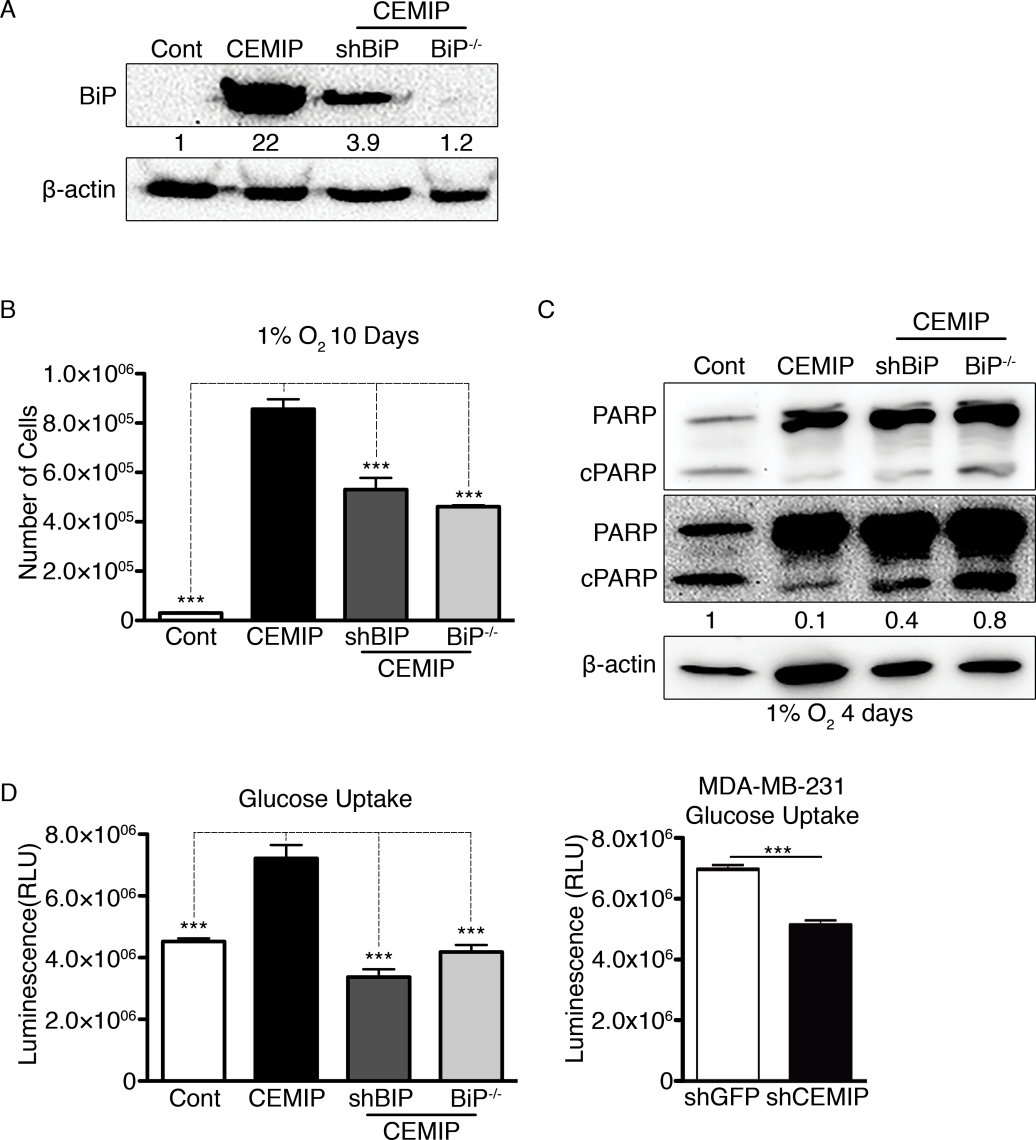
Upregulation of BiP contributes to CEMIP-mediated cell survival in hypoxic conditions and increased glucose uptake. MCF-7 Cont, MCF-7 CEMIP, MCF-7 CEMIP shBiP and MCF-7 CEMIP BiP^-/-^ cells were cultured under normoxic conditions except where otherwise noted. **(A)** Western blot analysis to confirm BiP knockdown or knockout in MCF-7 CEMIP shBiP and MCF-7 CEMIP BiP^-/-^ cells, respectively, using anti-BiP and anti-β-actin (loading control) antibodies. Densitometric analysis was performed to quantify protein bands. BiP levels were normalized to β-actin. **(B)** Number of cells in cultures that were plated in equal numbers and then counted after exposure to 1% O_2_ for 10 days. Mean ± SEM, ^***^p < 0.001, ANOVA test. **(C)** Western blot analysis of cells cultured for 4 days under hypoxic conditions. Two exposures of the blot probed with anti-PARP antibody are shown. β-actin was used as a loading control. Densitometric analysis was performed to quantify protein bands. Cleaved PARP levels were normalized to β-actin. **(D)** Glucose uptake in (left) genetically engineered MCF-7 cells and (right) MDA-MB-231 shGFP or MDA-MB-231 shCEMIP cells. Assays were initiated by adding 2-deoxyglucose and had an uptake time of 10 minutes. Mean ± SEM, ^***^p < 0.001, (left) ANOVA test and (right) Student’s t-test.

To investigate whether CEMIP mediates its pro-survival role in hypoxia by upregulating BiP expression, we produced MCF-7 CEMIP cell lines with reduced BiP expression using shRNA (named MCF-7 CEMIP shBiP cells) or CRISPR/Cas9 (referred to as MCF-7 CEMIP BiP^-/-^ cells) techniques. Western blotting confirmed that BiP expression is either reduced or completely ablated in the respective cell lines (Figure 4A). Next, we plated equal numbers of each cell line, allowed them to grow in 1% O_2_ for 10 days, and then performed cell counts. Reduced BiP expression in the MCF-7 CEMIP shBiP and MCF-7 CEMIP BiP^-/-^ cells rendered them more sensitive to hypoxia, as shown by the significantly lower number of cells relative to the MCF-7 CEMIP culture (Figure 4B). We found that the levels of cleaved PARP were modestly higher in MCF-7 CEMIP shBiP and MCF-7 CEMIP BiP^-/-^ cells than in parental MCF-7 CEMIP cells after 4 days in 1% O_2_ (Figure 4C). Our findings suggest that CEMIP-conferred resistance to hypoxic conditions is in part mediated by BiP, but additional mechanisms are probably involved.

It has been shown that BiP can upregulate glucose transporter 1 on the cell surface by facilitating its translocation [29]. Increased glucose uptake and metabolism mainly *via* aerobic glycolysis that confers resistance to hypoxia are hallmarks of cancer. We found that MCF-7 CEMIP cells exhibit higher glucose uptake than MCF-7 Cont cells, but glucose uptake in both MCF-7 CEMIP shBiP and MCF-7 CEMIP BiP^-/-^ cell lines was similar to the low level seen in MCF-7 Cont cells (Figure 4D). In contrast, MDA-MB-231 shCEMIP cells showed decreased glucose uptake as compared to MDA-MB-231 shGFP (Figure 4D). These results indicate that CEMIP expression promotes glucose uptake, and this effect is completely dependent on upregulation of BiP expression.

### Overexpression of CEMIP results in sustained tumor formation *via* BiP

**Figure 5:**
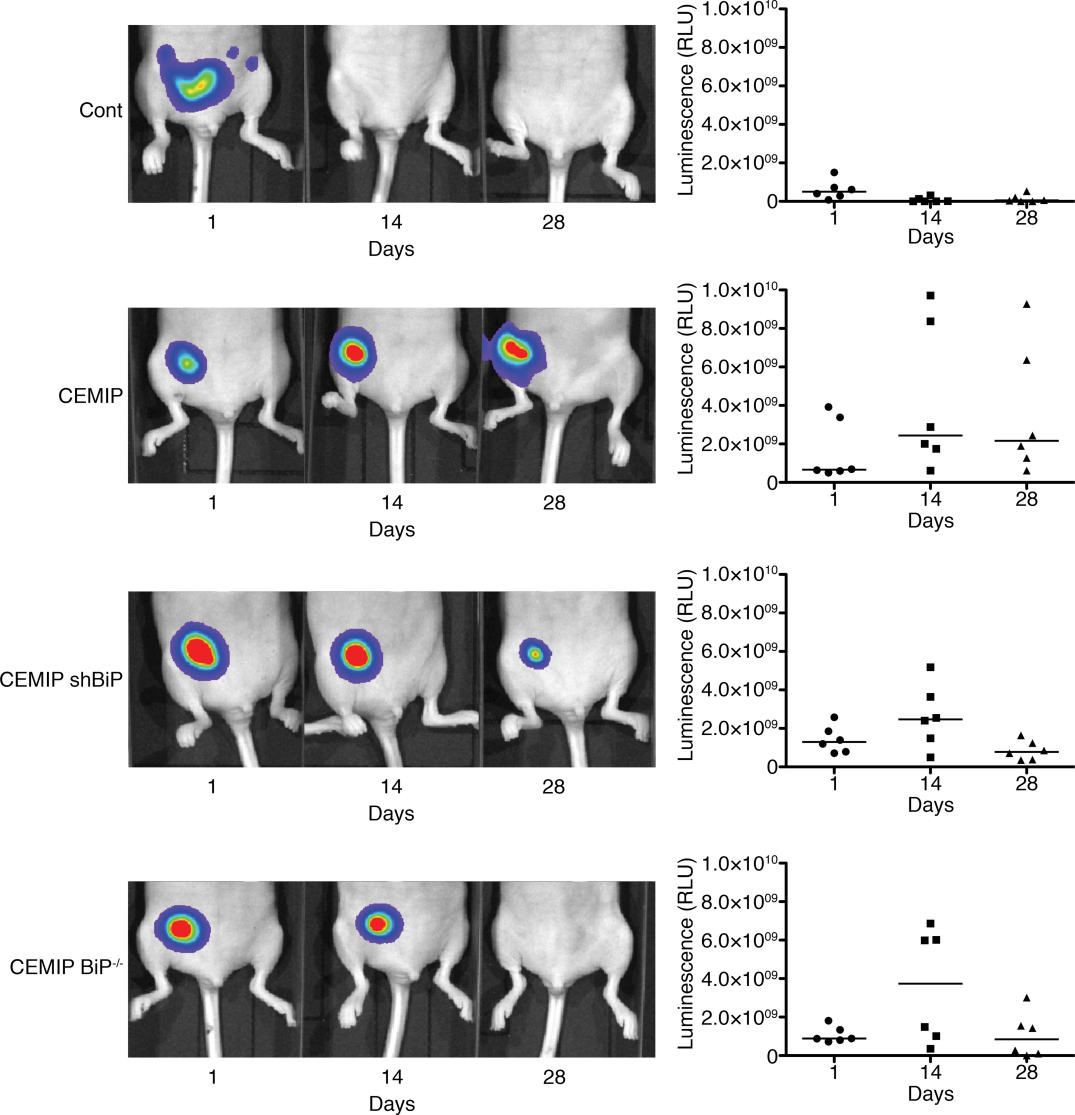
Overexpression of CEMIP in MCF-7 cells results in sustained tumor formation *via* BiP. MCF-7 Cont, MCF-7 CEMIP, MCF-7 CEMIP shBiP, and MCF-7 CEMIP BiP^-/-^ cells were transduced with a firefly luciferase-expressing lentivirus and implanted (5 x 10^6^ cells/mouse) in the mammary fat pad of female nude mice (n=6). Tumors were visualized using the IVIS imaging system at days 1, 14, and 28. Sequential images of one representative mouse from each group are shown. Graphs show quantified luciferase signals from each mouse.

We next investigated the consequences of CEMIP-mediated BiP upregulation on the growth of MCF-7 cells implanted in the mouse mammary fat pad. To allow *in vivo* imaging of the cells using an IVIS imaging system, we first stably expressed luciferase in our MCF-7 cell lines. It was previously demonstrated that MCF-7 cells do not form tumors when implanted in immunodeficient nude mice [30–32]. As expected, MCF-7 Cont cells did not grow in mice, and no tumors were detected by IVIS imaging 2 weeks after implantation (Figure 5, Supplementary Figure 7). Surprisingly, MCF-7 CEMIP cells formed tumors in the mouse mammary fat pad that persisted for at least 4 weeks (Figure 5, Supplementary Figure 7). In contrast, implanted MCF-7 CEMIP shBiP or MCF-7 CEMIP BiP^-/-^ cells formed tumors that grew for the first 2 weeks, followed by tumor regression by week 4 (Figure 5, Supplementary Figure 7). These results indicate that CEMIP expression promotes MCF-7 cell survival *in vivo*, and this effect is in part mediated by BiP upregulation.

## DISCUSSION

**Figure 6:**
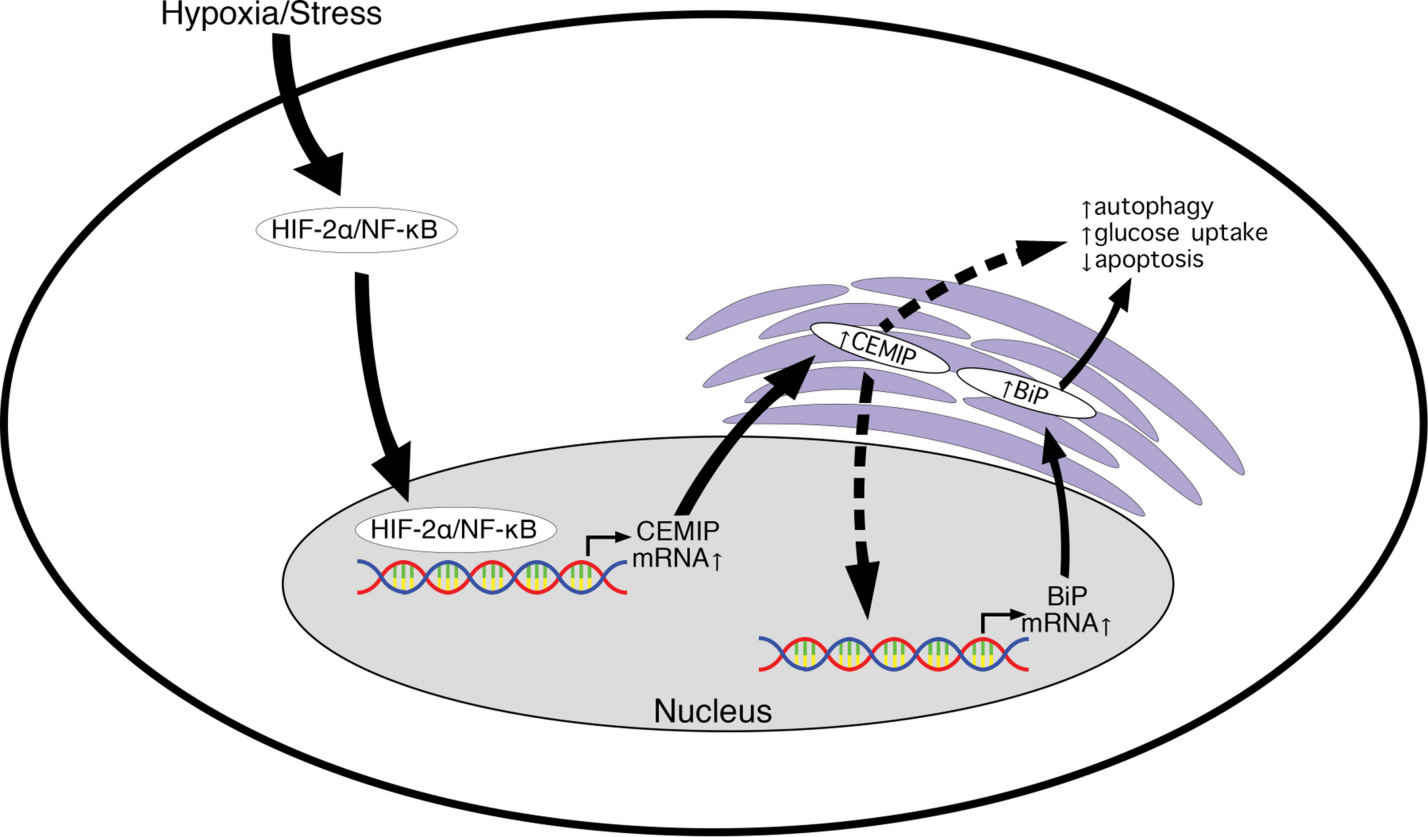
Model for CEMIP-mediated upregulation of BiP leading to increased cell survival during hypoxia/stress. HIF-2α and NF-κB accumulate in cells exposed to hypoxia or other stresses and bind directly to the CEMIP promoter. Increased CEMIP levels result in CEMIP-mediated activation of the BiP promoter by an unknown mechanism. Upregulation of BiP protein leads to activation of protective pathways such as increased autophagy and glucose uptake as well as decreased apoptosis. CEMIP is also activating another undetermined pro-survival mechanism independent of BiP.

This study identified for the first time a positive correlation between CEMIP and BiP expression in human breast cancer cell lines. We discovered that CEMIP upregulates BiP at the transcriptional level. We demonstrated this by overexpressing CEMIP in MCF-7 cells as well as by knocking down CEMIP in MDA-MB-231 cells. In both cell lines, CEMIP positively regulates BiP protein and transcript levels and BiP promoter activity. We also showed that cells overexpressing CEMIP exhibit reduced apoptosis and enhanced autophagic activity under hypoxia, which might support the ability of these cells to survive and proliferate under this stressful condition. Additionally, CEMIP upregulates glucose uptake. Together, these adaptive mechanisms might contribute to hypoxia resistance and allow CEMIP-overexpressing MCF-7 cells to form tumors that grow *in vivo* (Figure 6).

Our data indicate that CEMIP mediates at least some of its pro-survival effects through BiP. Reduced BiP expression in MCF-7 CEMIP cells, achieved by both shRNA and CRISPR/Cas9 techniques, rendered them more sensitive to hypoxia treatment. Although MCF-7 CEMIP shBiP and MCF-7 CEMIP BiP^-/-^ cells exhibited increased apoptosis under hypoxia as compared to parental MCF-7 CEMIP cells, MCF-7 Cont cells were still more sensitive to this condition. These findings suggest that while BiP contributes to CEMIP-mediated cell survival in hypoxia, there must be another mechanism facilitating this effect that is yet to be uncovered. One possible explanation would be that CEMIP exerts its pro-survival effects through EMT, a process that promotes tumor progression, as it’s been shown that CEMIP induces EMT [1, 2, 8, 14]. Additionally, reducing BiP expression in MCF-7 CEMIP cells decreased glucose uptake to a level similar to the one found in MCF-7 Cont cells, indicating that CEMIP promotes glucose uptake *via* BiP upregulation.

Several studies have reported that BiP is overexpressed in multiple cancers, where it plays an important role in carcinogenesis [22]. BiP expression in gastric cancer was found to be correlated with metastasis and negatively associated with patient survival [24]. Similar to our results using implanted MCF-7 CEMIP shBiP and MCF-7 CEMIP BiP^-/-^ cells, BiP knockdown in gastric and head and neck cancers resulted in decreased tumor growth and metastasis *in vivo* [20, 24]. BiP is known to exert an anti-apoptotic effect by binding directly to the pro-apoptotic protein BIK to block its downstream signaling and consequent cytochrome c release from mitochondria [33]. In addition, BiP has been shown to serve a protective role in the hypoxic microenvironment and to mediate chemoresistance [19, 22, 25]. However, the mechanisms by which BiP exerts these functions are still poorly understood.

Although BiP has been shown to be upregulated in hypoxic conditions, there is no evidence that it is a direct target of HIF-1/2. We show here that CEMIP activates the BiP promoter, but whether this transcriptional regulation occurs directly or indirectly needs further investigation. It was reported that transcriptional activation of BiP occurs mainly *via* an ER stress response element (ERSE), which contains multiple copies of the consensus sequence CCAAT(N9)CCACG upstream of the TATA box. Several transcription factors that activate ERSE, such as nATF6 (nuclear form of ATF6), NF-Y, TFII-I, and Sp1, have been identified [34–40]. BiP can also be induced independently of ERSE by an ATF4-CREB1 complex binding to an ATF4 site within the BiP promoter [41–43]. Since CEMIP has not been reported to act as a transcription factor, it is probable that it mediates regulation of BiP indirectly by activating one or more of the abovementioned transcription factors. CEMIP is more likely to affect ERSE-binding transcription factors, as our results show that CEMIP upregulates BiP transcription within a minimal promoter region (147 bp) that does not include the ATF4 binding site.

Activation of autophagy is one way in which CEMIP-mediated BiP upregulation can protect cells from apoptosis in hypoxia and contribute to tumor growth. BiP upregulation and consequent activation of the UPR is a well-established mechanism of autophagy activation [18, 19, 22]. UPR occurs when a cell stressor causes BiP to detach from three ER lumen proteins (PERK, IRE1 and ATF6), which causes their subsequent activation. PERK and IRE1 play crucial roles in inducing autophagy by activating key genes involved in this process. In the PERK response, eIF2α becomes phosphorylated, leading to activation of ATF4 and CHOP, which results in upregulation of autophagy genes such as LC3, ATG12, and ATG5. In the IRE1 pathway, JNK becomes activated and phosphorylates Bcl2, freeing it from a complex with Beclin1 and thus inducing autophagy [18, 19]. Our supplementary data (Supplementary Figure 6) show that the PERK and IRE1 arms of the UPR are activated in MCF-7 CEMIP cells exposed to hypoxia, further substantiating our findings.

The results presented in this study provide strong evidence that CEMIP upregulates BiP at the transcriptional level to mediate resistance to tumor microenvironments often characterized by oxygen and nutrient depletion. Our findings suggest that overexpression of CEMIP confers an adaptive advantage to cancer cells, which facilitates cell survival and tumor growth. Establishing the link between CEMIP and increased cell survival in adverse environmental conditions *via* BiP increases our understanding of how cancers progress.

## MATERIALS AND METHODS

### Materials

The following antibodies were used: PARP (#9542), BiP (#3177) and β-actin (#3700) from Cell Signaling Technology; CEMIP (#21129-1-AP) from Proteintech; Myc tag from Roche; LC3 (#PM036) from MBL International; and horseradish peroxidase-conjugated anti-mouse and anti-rabbit secondary antibodies from Rockland Immunochemicals. Bafilomycin A1 was acquired from Sigma-Aldrich. BiP CRISPR/Cas9 KO and HDR plasmids (sc-400073 and sc-400073-HDR, respectively) were purchased from Santa Cruz Biotechnology.

### Cell lines and culture

MCF-7, MDA-MB-231, and COS-1 cells were purchased from ATCC (Manassas, VA). Except where otherwise indicated, all cell lines were cultured in Dulbecco’s modified Eagle’s medium containing 10% fetal bovine serum and 100 IU/ml penicillin/100 μg/ml streptomycin in a humidified 5% CO_2_ in air atmosphere at 37°C and were used at early passage numbers. To achieve hypoxic conditions, cells were incubated at 37°C in a ProOx C21 hypoxia chamber (BioSpherix) under nitrogen with 1% O_2_ and 5% CO_2._ MCF-7 Cont and MCF-7 CEMIP stable cells were previously described [1]. MDA-MB-231 shGFP and MDA-MB-231 shCEMIP stable cells were generated by retroviral infection as previously described [1]. Briefly, the target cells were transduced with the retroviral supernatant obtained from GP2-293 packaging cells (Clontech) transiently co-transfected using polyethyleneimine (MW = 250,000 kDa, Polysciences, Inc.) with vectors encoding envelope gene or specific shRNA. Pooled stable cells were selected using puromycin. MCF-7 CEMIP cells were co-transfected with BiP CRISPR/Cas9 KO and HDR plasmids using Lipofectamine 3000 (Invitrogen) to generate BiP knockout cells named MCF-7 CEMIP BiP^-/-^. Pooled cells were selected using puromycin followed by fluorescence-activated single cell sorting to select cells expressing red fluorescent protein. MCF-7 CEMIP shBiP stable cells were produced using retroviral infection as described above.

### Western blotting

Cells were lysed in 2x Laemmli buffer, sonicated, and boiled. Whole cell lysates were resolved by SDS-PAGE followed by protein transfer onto nitrocellulose membranes for analyses using specific antibodies. Densitometric analysis of western blot data was performed using Image Lab (Bio-Rad) or AlphaView (ProteinSimple) software. For each lane, the observed experimental signal was normalized to the housekeeping protein (β-actin) signal.

### Quantitative real-time PCR (qRT-PCR)

RNA was extracted from cells using the RNeasy Kit (Qiagen) as per the manufacturer’s protocol, followed by quantitation using a NanoDrop ND-1000 spectrophotometer (Thermo Scientific). cDNA was generated using the Bio-Rad iScript cDNA Synthesis Kit, and qRT-PCR was performed using iQ SYBR-Green Super Mix (Bio-Rad) on an iQ5 RT-PCR machine (Bio-Rad). HPRT-1 was used as an internal control, and relative expression was calculated using the ΔΔCt method. Primers used were: HPRT-1, 5ʼ-ACCCCACGAAGTGTTGGATA (forward) and 5ʼ-AAGCAGATGGCCACAGAACT (reverse); CEMIP, 5ʼ-GCTCTTGAGTTGCATGGACA (forward) and 5ʼ-ACCGCGTTCAAATACTGGAC (reverse); and BiP, 5ʼ-GCTCGACTCGAATTCCAAAG (forward) and 5ʼ-TGACACCTCCCACAGTTTCA (reverse). Primers were synthesized by Operon (Huntsville, AL).

### DNA constructs

Expression constructs for Myc-tagged CEMIP and shRNAs targeting CEMIP or BiP and appropriate controls were previously described [1]. A 489 bp human BiP promoter sequence, obtained from DNA isolated from HT1080 cells, was cloned into pGL3-Basic (Promega), which contains a firefly luciferase reporter gene, using a PCR approach in the presence of 0.5 M GC-rich resolution buffer (Roche) as previously described [17]. 5’ truncations of the 489 bp BiP promoter were produced using the Q5 Site-Directed Mutagenesis Kit (New England Biolabs). Primers used to generate the reporter constructs are listed in the Supplementary Table 1. All constructs were confirmed by DNA sequencing.

### BiP promoter activity assay

MCF-7 and MDA-MB-231 cell lines were transiently co-transfected with the 489 bp BiP promoter luciferase reporter construct and a *Renilla* luciferase reporter plasmid (Promega) as an internal control using Lipofectamine 3000 (Invitrogen). COS-1 cells were transiently co-transfected with a BiP promoter luciferase reporter construct, Myc-CEMIP or empty vector as a control, and the *Renilla* luciferase reporter plasmid. DNA was incubated with polyethyleneimine (MW = 250,000 kDa, Polysciences, Inc.) for 30 minutes at room temperature before addition to the cells, and the medium was changed 18 hours later. 48 hours post-transfection, cells were lysed using 1X passive lysis buffer, and luciferase assays using the Dual-Glo Luciferase Assay System (Promega) were performed as recommended by the manufacturer. Luminescence was recorded using a SpectraMax L microplate reader (Molecular Devices). BiP promoter induction of firefly luciferase was normalized to the *Renilla* luciferase signal.

### Autophagic flux assay

Cells were cultured for 48 hours in 1% O_2_ and were treated with or without 100 nM bafilomycin A1 for the last 6 hours. Cell lysates were examined on western blots probed with antibody to LC3 to observe accumulation of LC3-II in the bafilomycin A1-treated cells.

### Transmission electron microscopy

Cells were cultured on mesh copper grids under hypoxia (1% O_2_) for 2 days and were then counterstained with uranyl acetate and lead citrate. Cells were observed under the transmission electron microscope (FEI Tecnai12 BioTwinG^2^) at 80 kV and images were taken using a CCD digital camera system (model XR-60, Advanced Microscopy Techniques Corp.).

### Cell proliferation assays

Equal numbers (180,000) of MCF-7 Cont and MCF-7 CEMIP cells were plated in 35 mm dishes and placed in the hypoxia chamber. Cells were collected at days 1, 3, 5, and 7, stained with trypan blue, and viable cells were counted. Equal numbers (50,000) of MCF-7 Cont, MCF-7 CEMIP, MCF-7 CEMIP shBiP, and MCF-7 CEMIP BiP^-/-^ cells were seeded in 6-well plates and cultured under hypoxia for 10 days, after which the cells were collected, stained with trypan blue, and viable cells were counted.

### Flow cytometry

Cells cultured under hypoxia (1% O_2_) for 6 days were collected, washed with PBS, and fixed with 70% ethanol for 15 minutes on ice. The cells were then washed and treated with propidium iodide:RNase A solution for 30 minutes. Flow cytometry analysis was performed using a BD FACSCalibur and at least 10,000 events were collected per sample. Data were analyzed using ModFit LT software.

### Glucose uptake

Glucose uptake was measured using the Glucose Uptake-Glo Assay (Promega) according to the manufacturer’s protocol. Briefly, 30,000 cells were plated and initiated with 1 mM 2-deoxyglucose for 10 minutes the following day.

### mRNA expression analysis in breast cancer cell lines

Analysis of data in the Cancer Cell Line Encyclopedia (Novartis/Broad, Nature 2012) was performed using cBioPortal. CEMIP mRNA expression z-scores from 51 breast cancer cell lines contained within the dataset were used to identify cell lines that express high (z-score > 0.6) or low (z-score < -0.3) CEMIP mRNA levels. BiP mRNA expression z-scores of cells in each population were then determined.

### Orthotopic tumor implantation and imaging in mice

Cells for implantation were infected with lentivirus expressing firefly luciferase (Cellomics Technology), followed by blasticidin selection. Cells were collected by trypsinization, washed twice with PBS, and mixed with Matrigel (final concentration of 4 mg/ml, Corning). 5 x 10^6^ cells per mouse in 50 μl were orthotopically injected into the mammary fat pad of female athymic nude mice (Envigo, Hsd:Athymic Nude-*Foxn1^nu^*). On days 1, 14 and 28 post-implantation the mice were anesthetized using inhaled isoflurane (1-2%), injected intraperitoneally with 150 μl RediJect D-Luciferin (PerkinElmer), and 10 minutes later the tumors were visualized using the IVIS Lumina III imaging system (PerkinElmer). IVIS images were quantified using Living Image v4.3.1 software (PerkinElmer). Experiments using mice were conducted in accordance with the Office of Laboratory Animal Welfare and approved by the Institutional Animal Care and Use Committee of Stony Brook University.

### Statistical analysis

Data are expressed as the mean ± SEM of triplicates. Each experiment was repeated at least 3 times. Student’s t-test or ANOVA were used to evaluate differences using GraphPad Prism software. *, p < 0.05; **, p < 0.01; ^***^, p < 0.001.

## SUPPLEMENTARY MATERIALS FIGURES AND TABLE



## Abbreviations

BiP: binding immunoglobulin protein
CEMIP: cell migration-inducing protein
EGFR: epidermal growth factor receptor
EMT: epithelial-to-mesenchymal transition
ER: endoplasmic reticulum
ERSE: ER stress response element
HIF-2α: hypoxia-inducible factor-2
NF-κB: nuclear factor kappa-light-chain-enhancer of activated B cells
TEM: transmission electron microscopy
UPR: unfolded protein response
