# Supplementary material for: CEMIP upregulates BiP to promote breast cancer cell survival in hypoxia
Source: Oncotarget. 2019 Jul 2;10(42):4307–20. doi: 10.18632/oncotarget.27036 (PMC6611512; doi:10.18632/oncotarget.27036)
Supplement: Supplementary file 1 [file oncotarget-10-4307-s001.pdf]

CEMIP upregulates BiP to promote breast cancer cell survival in hypoxia

SUPPLEMENTARY MATERIALS

Anti-PDI, anti-phospho-eIF2α, and anti-BECN1 antibodies were purchased from Cell Signaling Technology. Anti-Calnexin antibody was purchased from Stressgen. Anti-phospho-IRE antibody was purchased from Novus Biologicals.

Adenovirus expressing CEMIP and GFP was purchased from Vector Biolabs and adenovirus expressing GFP was produced as previously described [1].

EGFR construct was acquired from Addgene and Timp1 construct was produced as previously described [2].

REFERENCES

1. Ballou, L.M., et al., Dual regulation of glycogen synthase Kinase-3 by the 1A-Adrenergic receptor. J Biol Chem, 2001. 276: p.40910-6. https://doi.org/10.1074/jbc.M103480200. [PubMed].
2. Cao, J., et al., Membrane type matrix metalloproteinase 1 activates pro-gelatinase A without furin cleavage of the N-terminal domain. J Biol Chem, 1996. 271: p.30174-80. https://doi.org/10.1074/jbc.271.47.30174. [PubMed].

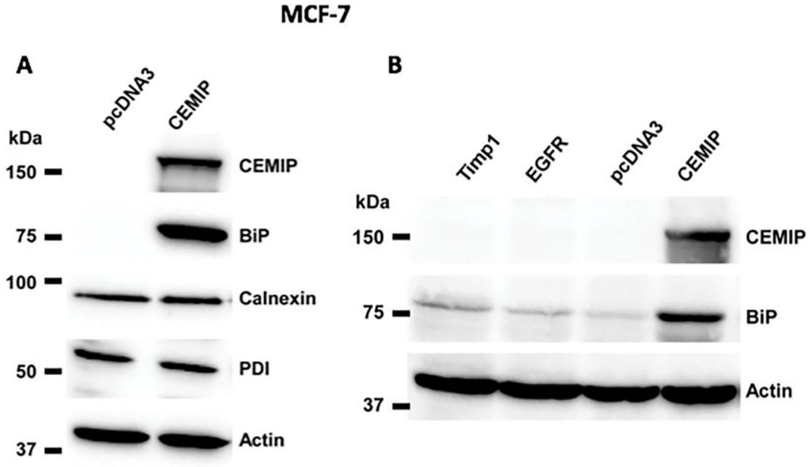

Supplementary Figure 1: CEMIP upregulation of BiP is specific and not an artifact caused by overexpression of an exogenous protein. (A) MCF-7 cells were transiently transfected with CEMIP or empty vector as a control. After 48 hours, cells were lysed and analyzed by western blotting. Actin serves as a loading control. CEMIP upregulated BiP and did not affect the other ER chaperones. **(B)** MCF-7 cells were transiently transfected with CEMIP, EGFR, Timp1 or empty vector as a control. After 48 hours, cells were lysed and analyzed by western blotting. BiP upregulation was caused by overexpression of CEMIP but not the other proteins.

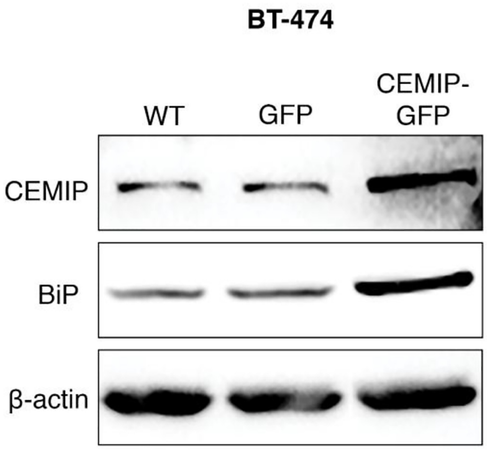

Supplementary Figure 2: CEMIP upregulates BiP in BT-474 human breast cancer cells. BT-474 cells were left untreated (WT) or were infected with adenoviruses expressing GFP or CEMIP-GFP. 48 hours post-infection, cells were lysed and analyzed by western blotting. β-actin serves as a loading control.

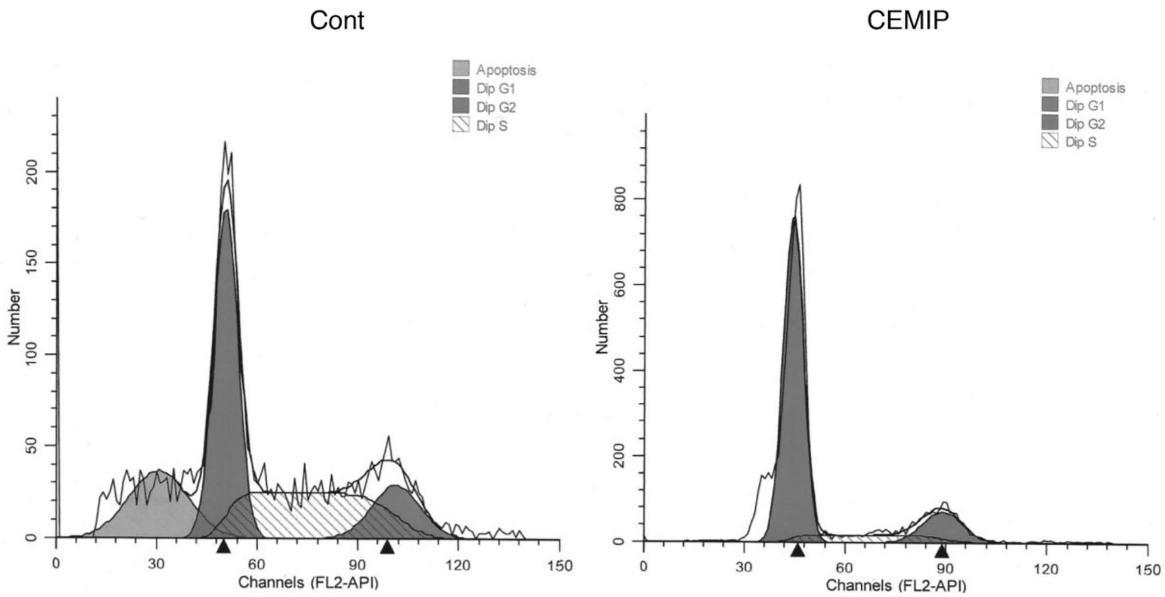

Supplementary Figure 3: CEMIP overexpression protects MCF-7 cells from apoptosis in hypoxia. MCF-7 Cont and MCF-7 CEMIP cells were cultured under hypoxic conditions for 6 days, then stained with propidium iodide and subjected to flow cytometry. Histograms represent cell cycle distribution analyzed by ModFit LT software. The data show that there are fewer apoptotic cells in the MCF-7 CEMIP culture.

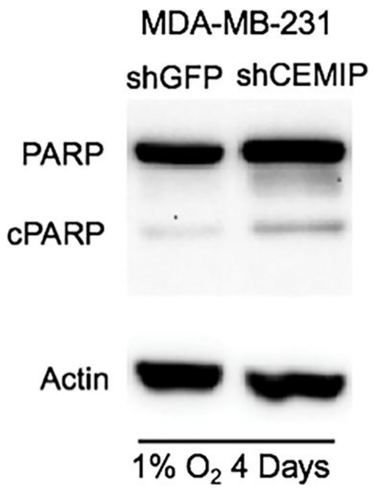

Supplementary Figure 4: CEMIP protects MDA-MB-231 cells from apoptosis in hypoxia. MDA-MB-231 shGFP and MDA-MB-231 shCEMIP cells were cultured under hypoxia for 4 days. Cell lysates were subjected to western blotting to detect full-length PARP and cleaved PARP (cPARP). Actin serves as a loading control. Increased PARP cleavage is seen in the CEMIP knockdown cells.

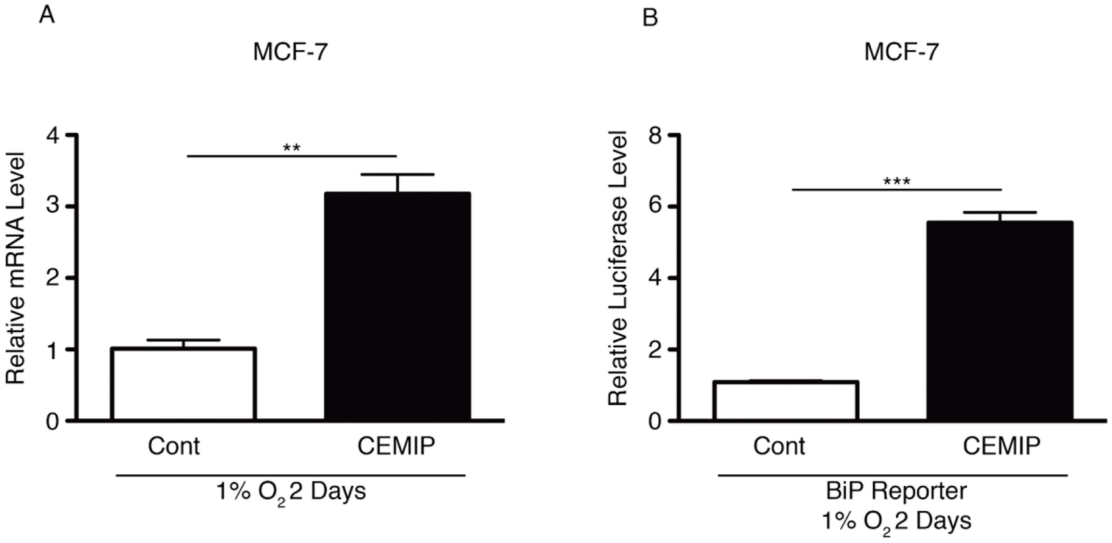

Supplementary Figure 5: CEMIP upregulates BiP transcription in MCF-7 cells in hypoxia. MCF-7 Cont or MCF-7 CEMIP cells were cultured in hypoxic conditions for two days. **(A)** Relative BiP mRNA levels were determined by qRT-PCR. BiP expression was normalized to HPRT-1. Mean ± SEM, $**p < 0.01$, Student's t-test. The results demonstrate a positive correlation between CEMIP expression and BiP transcript levels. **(B)** Relative firefly luciferase reporter activity in cells transfected with the 489 bp human BiP promoter reporter construct. Firefly luciferase reporter activity was normalized to the *Renilla* luciferase signal. Mean ± SEM, $***p < 0.001$, Student's t-test. The results reveal that CEMIP increases BiP promoter activity in hypoxia.

Supplementary Table 1: Primers used to generate reporter constructs

Reporter constructs	Forward (5 to 3)	Reverse (5 to 3)
489 bp	ATGGTACCGGGTGAGGGATGGAGGAAG	ATAGATCTCGAAACACCCCAATAGGTCA
380 bp	ATGGTACCGAGTGAAGGCGGGACTTGT	ATAGATCTCGAAACACCCCAATAGGTCA
285 bp	ATGGTACCCAATGAACGGCCTCCAAC	ATAGATCTCGAAACACCCCAATAGGTCA
147 bp	ATGGTACCGATGGGGCGGATGTTATCTA	ATAGATCTCGAAACACCCCAATAGGTCA

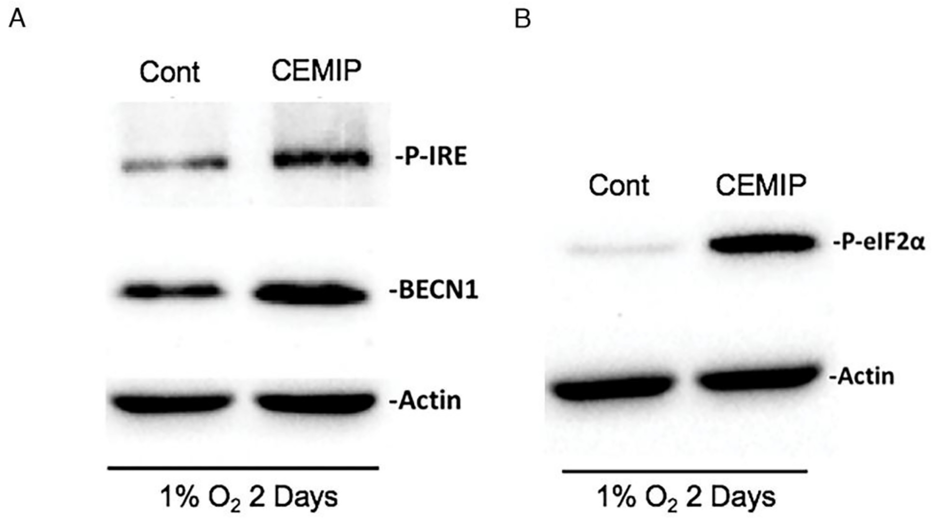

Supplementary Figure 6: Increased activity of the IRE1 and PERK arms of the UPR in CEMIP-overexpressing cells grown under hypoxia. MCF-7 Cont and MCF-7 CEMIP cells were subjected to hypoxia treatment for 2 days. Cells were lysed and analyzed by western blotting. Actin serves as a loading control. **(A)** Phospho-IRE and protein levels of its downstream signaling target BECN1 are higher in MCF-7 CEMIP cells than MCF-7 Cont cells. **(B)** Phospho-eIF2α, a downstream target of phospho-PERK, is higher in MCF-7 CEMIP cells than MCF-7 Cont cells.

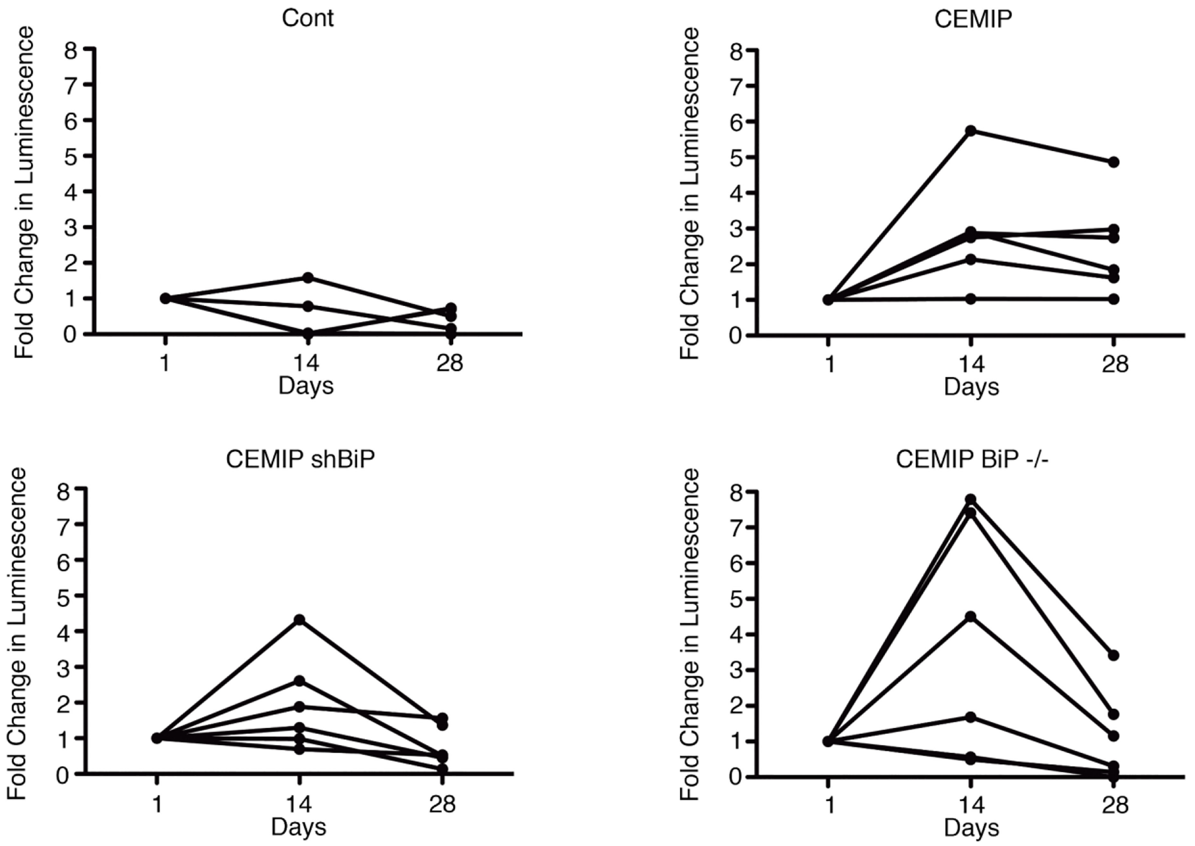

Supplementary Figure 7: Overexpression of CEMIP in MCF-7 cells results in sustained tumor formation. Luciferase-expressing MCF-7 Cont, MCF-7 CEMIP, MCF-7 CEMIP shBiP and MCF-7 CEMIP BiP$^{-/-}$ cells (5 x 10^6 cells/mouse) were implanted in the mammary fat pad of female nude mice (n=6). IVIS images were obtained at days 1, 14, and 28 and the luciferase signals were quantified using Living Image software. The signals for each mouse were normalized to the value at day 1. In the MCF-7 Cont group, 3 lines are not visible as they overlap. The results show sustained tumor formation in the MCF-7 CEMIP group, while tumors in the MCF-7 Cont group did not grow. Most of the implanted MCF-7 CEMIP shBiP and MCF-7 CEMIP BiP$^{-/-}$ tumors grew larger but regressed by day 28.
